# The Role of Mitochondrial DNA (mtDNA) in the Development of Diabetic Retinopathy (DR): A Systematic Review

**Published:** 2017

**Authors:** Negar Sarhangi, Fatemeh Khatami, Abbasali Keshtkar, Ramin Heshmat, Rasha Atlasi, Mahsa Mohammadamoli

**Affiliations:** 1 Endocrinology and Metabolism Research Center, Endocrinology and Metabolism Clinical Sciences Institute, Tehran University of Medical Sciences, Tehran, Iran; 2 Chronic Diseases Research Center, Endocrinology and Metabolism Population Sciences Institute, Tehran University of Medical Sciences, Tehran, Iran; 3 Metabolomics and Genomics research Center, Endocrinology and Metabolism Molecular- Cellular Sciences Institute, Tehran University of Medical Sciences, Tehran Iran; 4 Department of Health Sciences Education Development, School of Public Health, Tehran University of Medical Sciences, Tehran, Iran

**Keywords:** DNA, Mitochondrial, Diabetic Retinopathy, Polymorphism, Genetic, Mutation

## Abstract

Diabetic Retinopathy (DR) is the most prevalent health problem, which is influenced by environmental and genetic factors with an increasing prevalence. The current systematic review is focused on mtDNA modification, including polymorphism and mutation/deletion, with a direct effect on DR.This systematic search was initially done through PubMed, Cochrane, EMBASE, SCOPUS, and Web of Science without a restriction on the years of publication. The terms searched included ‘‘mtDNA’’, ‘‘mitochondrial DNA’’, ‘‘diabetes’’, ‘‘diabetic’’, ‘‘retina’’, and ‘‘diabetic retinopathy’’. Animal, cohort, cross-sectional, and in vitro studies, as well as case series, case reports, review articles, and Letters to Editor were excluded from this research.From 1528 resulting searched articles, only 12papers were finally chosen as the case-control studies considering mtDNA gene and DR. Actually, of these 12 articles, 8 studies were concerned with mtDNA polymorphisms (UCP1, UCP2, ROMO-1, and Mn-SOD) and 4 articles were related to mtDNA mutation (A3243G mutation in tRNA^Leu(UUR) ^gene and mtDNA deletion (ΔmtDNA 4977)).Some conflicting results were found between the selected genetic modifications of mtDNA, such as Mn-SOD, UCP1, ΔmtDNA 4977, tRNALeu (UUR), and ROMO-1.Finally, A3243G mutation in the tRNA^Leu (UUR) ^gene and rs660339 and V16A polymorphisms of UCP2 and Mn-SOD genes were respectively considered as the most important factors in the pathogenesis of DR.

## INTRODUCTION

As a major public health problem, diabetes became an epidemic disease in the 21^st^ century. The functions of different organs are influenced by the disease, which either reduces life quality or leads to death in some cases [1, 2]. An increased circulation of blood glucose through blood vessels is induced by this lifelong disease, which results in varied micro- and macro-vascular complications. Though preventable in the developed countries, Diabetic Retinopathy (DR) as one of the most severe types of these complications mainly leads to blindness in the vision-disabled elderly [2-5]. An increasing prevalence of diabetes has been reported by the International Diabetes Federation (IDF). Approximately, one-third of diabetic people (93 out of 246 million people) estimated for DR symptoms, whom 17 and 28 million people of them have developed Proliferative Diabetic Retinopathy (PDR) and vision-threatening conditions like Diabetic Macular Edema (DME), respectively [1]. DR develops at a slow rate of progression in such a way that involved patients show no symptoms at the beginning and when they began to experience a deteriorating eye sight, they have been already involved in an advanced level of retinopathy, which can then lead to the loss of vision if not controlled [3]. Early detection of the disease and its treatment are important for improving the diabetic patients’ qualities of life [6]. In addition to hyperglycemia, which is the principal cause of DR progression, hypertension and dyslipidemia are some other risk factors associated with the disease [4, 7]. The exact mechanism of the pathogenesis of DR is not clear. A cascade of biochemical and physiological changes that finally cause micro-vascular impairment and retinal dysfunction leads to various clinical conditions[4]. Several biochemical pathways are involved in the increasing amounts of sorbitol and Advanced Glycation End-products (AGEs), oxidative stress, protein kinase C activation, inflammation, and angiogenesis induced by Vascular Endothelial Growth Factor (VEGF), which modulates the pathogenesis of retinopathy [8]). Under the influences of many molecular, biochemical, and functional abnormalities, the retinal mitochondria become dysfunctional in DR [3, 9], while their copy numbers are reduced and DNAs (mtDNAs) are damaged [10]. Mitochondrial DNA (mtDNA) is a double-stranded circular molecule with 16,569 base pairs length, which is packed as nucleoid-like structures unlike nuclear DNA with introns and nucleosomes, lacks protective histones, and has a non-coding region and Displacement loop (D-loop) that contains essential transcription and replication elements. Hence, it is 10-20 times more susceptible to oxidative impairment than nuclear DNA [10-13]. The damage is more extensive at the D-loop than at any other regions of the mtDNA[14]. Decreased copy numbers of mtDNA and transcriptions of mtDNA-encoded genes, which play a vital role in the functioning of the electron-transport system occur [6]. Superoxide levels are elevated and the enzymeMnSOD is responsible [15-17]. Also, initializations of the apoptotic machinery and capillary cell apoptosis proceed the development of retinal histopathology associated with DR [9, 18,19]. Besides the metabolic and physiologic factors, the pathogenesis of DR is affected by genetic factors. The risk of severe DR in the patient’s siblings is almost three times higher, while diabetic patients with the same risk factors are involved in varying severities of retinopathy [20, 21]. Some cohort studies indicated the fact that mtDNA variations are associated with the severity but not presence of DR [22, 23]. Some data reveal that the effects of mtDNA variations like Single Nucleotide Polymorphism (SNP) and genetic mutations, as well as ischemia or neovascularization are related to the features of PDR. There are a wide range of studies, in which a correlation between mtDNA gene modification and DR has been found. Most of them are review studies, but none of them is a systematic review [24-26]. Thus, the current research was conducted to represent a collection of all analytical observational studies related to the mtDNA mutations and SNPs in a systematic review. In fact, the aim of our study was to review the importance of mtDNA variations and the risk of DR.

## MATHRIALS AND METHODS

**Data Sources and Searches**

This study was developed according to Preferred Reporting Items for Systematic Reviews and Meta-Analyses (PRISMA) guidelines. After receiving Prospero code (CRD42017058832), a computerized search was done in the relevant literature to retrieve the evidence on the topic by using the databases of Cochrane, PubMed, EMBASE, SCOPUS, and Web of Science, as well as keywords of ‘‘mtDNA’’, ‘‘mitochondrial DNA’’, ‘‘diabetes’’, ‘‘diabetic’’, ‘‘retina’’, and ‘‘diabetic retinopathy’’ without any limitations on the years of publication. To perform a comprehensive search, different combinations of the mentioned terms were utilized. The titles and abstracts of all the search results were examined to eliminate the duplications and multiple publications from the same research, while the most relevant data were employed for the investigation. Also, to find any additional publications related to the topic, the lists of references included in the papers were searched manually.

**Selection of the Studies**

For assessing the association between mtDNA and DR, we included all the observational studies performed as case-control and cross-sectional studies only on humans. Any additional abstracts or unpublished studies like those carried out in vitro or on animals, theses or dissertations, short communications, reports, Letters to Editor, patents, and reviews or mini-reviews were excluded from the current systematic review. Also, the titles and abstracts of the publications were screened to exclude any irrelevant findings. Moreover, 2 reviewers (FKH and NS) evaluated the eligible articles based on the inclusion and exclusion criteria by independently reviewing their titles and abstracts. Then, to find the relevant publications to the topic, the same reviewers fully assessed the texts. Any cases of conflicts were supervised by a third reviewer (MMA) for selecting and screening the studies.

**Data Extraction**

The data for each study were extracted by using a standardized data extraction form, which was designed to include the following information in an Excel sheet: Patient’s characteristics (sample size, age, and sex), study design, method of analysis, author(s), year, polymorphism and mutation, city, ID number, and significant association.

## RESULTS

**Figure 1:**
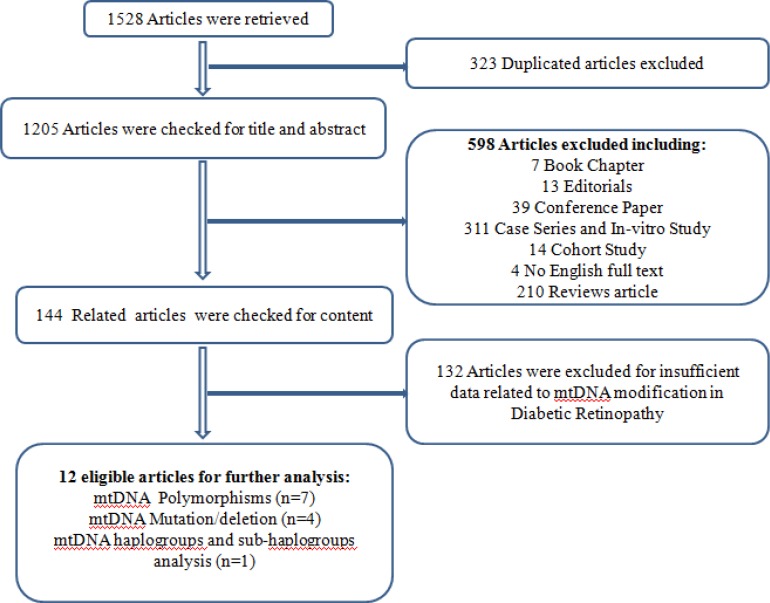
A Flow Diagram Presenting the Results of the Literature Search and Selection Processes of the Studies

Most researches were in-vitro studies and review articles. 144papers were investigated for content and only 12 relevant articles were finally selected as an additional step. Of these 12 articles (3256 vs. 2910), 8 and 4 studies were concerned with mtDNA polymorphisms and mtDNA mutations and deletions, respectively (Fig 1).

Totally, different polymorphisms and mutations of the following genetic variants and mutations were found in the selected studies (Table1):

Thermogenin (uncoupling protein 1 or UCP1) is an uncoupling protein found in the mitochondria of Brown Adipose Tissue (BAT). Mitochondrial uncoupling proteins (UCP) are members of the larger family of Mitochondrial Anion Carrier Proteins (MACP). UCPs separate oxidative phosphorylation from ATP synthesis with energy dissipated as heat. UCPs smooth the progress of anion transfer from the inner to the outer mitochondrial membrane and proton insertion from the outer to the inner mitochondrial membrane. Their role is to produce heat through non-shivering thermogenesis.

 Mitochondrial uncoupling protein 2 (UCP2) is a protein that is encoded by UCP2 gene in humans. It has a role in H+/OH- transfer. This gene is expressed in many tissues with a maximum expression in the skeletal muscle. Although it has been originally thought to play a role in no-shivering thermogenesis, obesity, diabetes, and atherosclerosis, it now appears that the main function of UCP2 is to control mitochondria-derived Reactive Oxygen Species (ROS).

Manganese superoxide dismutase (Mn-SOD) in the mitochondrial antioxidant defense system whichis completely activated in the mitochondria to detoxify the oxygen free radicals generated by mitochondrial respiration. Thus, oxidative stress leads to the up-regulation of Mn-SOD gene whose activity can be further enhanced by the Mitochondrial Protein Influx (MPI).

 Reactive oxygen species modulator 1 (Romo1) is a mitochondrial membrane protein responsible for increasing ROS levels in cells. The protein also has antimicrobial activities against a variety of bacteria by inducing bacterial membrane breakage.

 The common mitochondrial DNA4977-bp deletion (mtDNA 4977) often happens in the tissues of high oxygen demands and low mitotic activities, including the brain, heart, and skeletal muscle.

MT-TL1 gene encoding tRNAleucine 1, tRNA^Leu(UUA/UUG)^, is designated as mitochondrial tRNA^Leu (UUR)^gene. This molecule inserts theleucine (Leu) into proper locations during protein growth after attaching to it. It is present in cellular structures like mitochondrial structure and converts food energy into a usable form of energy for cells. It further participates in the mitochondrial protein assembly that contributes to the oxidative phosphorylation process, through which oxygen, simple sugars, and fatty acids are mixed to make adenosine triphosphate (ATP) as the main source of energy for cells.

**Table1:** Characteristics of the Selected Studies on mtDNA and DR

**First Author**	**Title**	**Country**	**Year**	**Cases/Controls**	**Mean age of Case**	**Mean age of Controls**	**mtDNA Modification**	**Methods**
**Alessandro Achilli[** **25** **]**	Mitochondrial DNA Backgrounds Might Modulate Diabetes Complications Rather than T2DM as a Whole	Italy	2011	466/438	65.8468.19	59.9669.97	57 different mtDNAhaplogroups and sub-haplogroups	Sequencing of entire mtDNA genomes
**Daisy Crispim[** **26** **]**	Polymorphisms of the UCP2 gene are associated with proliferative diabetic retinopathy in patients with diabetes mellitus	Brazil	2010	325/369	50.2	46.8	Polymorphism (866G/A, Ala55Val and Ins/Del polymorphisms in the UCP2 gene)	RFLP Real time PCR
**Masataka Fukuda [** **27** **]**	Mitochondrial DNA Mutations Are Associated with Both Decreased Insulin Secretion and Advanced Microvascular Complications in Japanese Diabetic Subjects	Japan	1999	537/612	60.3 ± 0.6	49.5 ± 0.7	mtDNA mutation (eight kinds of Mt-DNA point mutations and all of which were single mutations were found in 74 subjects)	PCR-RFLP (Digestion)
**S.F.Haghighi[** **28** **]**	Polymorphic Variant of MnSODA16V and Risk of Diabetic Retinopathy	Iran	2014	140/140	52.5 ± 22.5	52.5 ± 22.5	Polymorphism (MnSODA16VC47T)	PCR-RFLP
**Seong Jin Lee [** **29** **]**	Association of manganese superoxide dismutase gene polymorphism (V16A) with diabetic macular edema in Korean type 2 diabetic patients	Korea	2006	304/192	53.4 ± 13.3	52.1 ± 13.2	Polymorphism (V16A polymorphism of the Mn-SOD and polymorphic region of the ACE gene)	PCR-RFLP
**MojcaGloboˇcnikPetroviˇ [** **30** **]**	Manganese superoxide dismutase gene polymorphism (V16A) is associated with diabetic retinopathy in Slovene (Caucasians) type 2 diabetes patients	Slovenia	2008	283/143	66.0 ± 9.5	66.9 ± 11.5	Polymorphism (V16A polymorphism of the Mn-SOD gene)	PCR-RFLP
**MojcaGlobocnikPetrovic[** **31** **]**	The rs6060566 of the reactive oxygen speciesmodulator 1 (Romo-1) gene affects Romo-1expression and the development of diabeticretinopathy in Caucasians with type 2 diabetes	Slovenia	2015	278/528	64.9 ± 8.0	63.8 ± 9.0	Polymorphism (rs6060566 of the ROMO-1 gene)	fluorescence-based competitive allelespecificPCR (KASPar) assay
**YinchenShen[** **32** **]**	Investigation of Variants in UCP2 in Chinese Type 2 Diabetes and Diabetic Retinopathy	China	2014	324/155	58.15 ±12.52	60.15 ±10.90	Polymorphism (rs660339 in the exon 4 and rs659366 in promoter region of UCP2 gene)	sequencing
**VanitaVanita[** **33** **]**	Association of RAGE (p.Gly82Ser) and MnSOD (p.Val16Ala) Polymorphisms with Diabetic Retinopathy in T2DM patients from North India	India	2014	446/312	55.92 ± 8.90	55.84 ± 12.2	Polymorphism (Gly82Ser (RAGE) and Val16Ala (MnSOD))	sequencing
**Yue Zhang [** **34** **]**	The gene polymorphisms of UCP1 but not PPAR c and TCF7L2 are associated with diabetic retinopathy in Chinese type 2 diabetes mellitus cases	China	2015	448/344	62.35 ± 11.92	60.16 ± 11.67	polymorphisms (rs1800592 in the UCP1 gene, rs1801282, rs3856806 and rs1249719 in the PPARc gene and rs11196205 in the TCF7L2 gene)	PCR-LDR (polymerasechain reaction-ligase detection reactions)
**D.J. Holmes-Walker [** **35** **]**	Does Mitochondrial Genome Mutation in Subjects with Maternally Inherited Diabetes and Deafness Decrease Severity of Diabetic Retinopathy?	Australia	1998	18/11	42.1 6 13.8	42.36 14.9	The 3243 mutation 3243 A-G tRNA^LEU (UUR) ^point mutation of mitochondrial DNA	-
**Susumu Suzuki [** **14** **]**	Oxidative damage to mitochondrial DNA and its relationship to diabetic complications	Japan	1999		22/7	-	mtDNA deletion of 4977 bp (DmtDNA4977)	Quantification of mtDNA4977 with ompetiti6e PCR

## DISCUSSION

DR is a common life-affecting micro-vascular complication of diabetes and the main cause of new cases of blindness troubling adult patients with diabetes mellitus [36]. Diabetes has such a complex and wide-ranging pathophysiology that necessitates numerous studies to be focused on finding its genetic modifications [37-43]. It has been shown that increasing oxidative stress in the retinas of diabetic patients leads to the escalation of oxidative modified DNA [44]. Augmented oxidative stress through hyperglycemia possibly contributes to the pathogenesis of diabetic complications with aDNA marker of 8-hydroxydeoxyguanosine (8-OHdG), which is coupled with mtDNA deletions [11]. mtDNA 4977-bp (ΔmtDNA4977) deletion and 8-OHdG content in the muscle DNA of Non-Insulin-Dependent Diabetes Mellitus (NIDDM) patients have been found to be actually much higher than those of the control subjects [11]. Moreover, ΔmtDNA4977 and 8-OHdG content are interrelated with the duration and severity of diabetic nephropathy and retinopathy[11]. The functions of antioxidants in the management of diabetes and its complications have been studied several times [45, 46]. The rs6060566 polymorphism of Romo-1 gene is linked with DR development [33]. Besides, the rs6060566 polymorphism can be mediated by the expression of Romo-1 gene in the retina [33]. There is some evidence that the genetic variation of mtDNA contributes to the pathogenesis of DR. mtDNA modification includes the genetic variants (polymorphisms) of a common population, by which human population is separated into somatic mutations of mtDNA[47] and mitochondrial copy number changes as a result of Displacement-loop (D-loop) [48, 49]. Diabetes complications developed by important mtDNA variations are usually classified into micro-vascular complications, including diabetic nephropathy, neuropathy, and retinopathy and macro-vascular complications, such as cardiovascular diseases, cerebrovascular accidents, and peripheral vascular diseases [50, 51]. Interestingly, SNP rs1800592 of UCP1, but not peroxisome proliferators-activated receptor-gamma (PPAR*γ*) and Transcription factor7-like2 (TCF7L2) has been found to be coupled with DR in a sample of Chinese patients [52]. The two important mtDNA genes in the pathogenesis of DR are uncoupling proteins 1 and 2 (UCP1 and UCP2) with the main role of controlling ROS. UCP2 separates the substrate oxidation from ATP synthesis and thus reduces ATP production by the mitochondrial respiratory chain [53-55]. It has been checked whether rs659366, rs660339, and 45-bp Insertion/Deletion (Ins/Del) polymorphisms in the UCP2 gene may be associated with PDR [28, 34]. The rs660339 has been found as an important risk factor associated with DR in both type 1 and 2diabetes groups [28].

Moreover, the protective effect of magnesium on the renal functions of diabetic rats has been shown [55-57]. In fact, Mn-SOD is a mitochondrial antioxidant enzyme located in the cellular defense system against the agents that induce oxidative stress [58, 59]. Mn-SOD A16V (C47T) polymorphism has been shown to be associated with the risk of DR[30]. Evaluation of the V16A polymorphism of the Mn-SOD gene during diabetes development has shown an association between its VV genotype and DR in Caucasians with type 2 diabetes [32]. Hence, the mentioned polymorphism can provide a genetic marker of DR in Caucasians [32]. These conflicting results revealed that the V16A polymorphism of the Mn-SOD gene is not connected to the development of diabetes and progression of DR, but is associated with DME [31]. These findings were found to be in agreement with the results of another case-control study that evaluated the association of the Receptor for Advanced Glycation End-products (RAGE) (p.Gly82Ser) and Mn-SOD (p.Val16Ala) polymorphisms with DR in diabetic patients. The findings indicated a statistically significant association only between RAGE Gly82Ser polymorphism and DR [35]. The most frequent mtDNA mutation for diabetes is the A3243G mutation in the tRNA^Leu (UUR) ^gene [60, 61]. The sequencing results of an mtDNA fragment surrounding the tRNA^Leu (UUR) ^site suggested that mtDNA mutations in Japanese diabetic subjects are completely correlated with diabetes and advanced diabetic micro vascular complications [29]. Additional studies have demonstrated that abnormal glucose tolerance raises the clinical expression of pigmentary retinopathy in patients with a mitochondrial genome mutation [62]. Due to the heterogeneity of the selected mtDNA variations in this paper, a meta-analysis was not followed in our qualitative systematic review. By way of illustration, the genetic variations were completely different both in the candidate genes and selected polymorphisms with varied impacts on the gene expression profile.

## CONCLUSIONS

Taking everything into consideration, the importance of mitochondria in DR was found to be hidden in the variations in its genes like A3243G mutation in the tRNA^Leu (UUR) ^gene, and ΔmtDNA4977, Mn-SOD A16V (C47T), UCP1, UCP2, and ROMO-1 polymorphisms.
